# Place of the partial dopamine receptor agonist aripiprazole in the management of schizophrenia in adults: a Delphi consensus study

**DOI:** 10.1186/s12888-022-04008-9

**Published:** 2022-05-28

**Authors:** Pierre-Michel Llorca, Philippe Nuss, Éric Fakra, Isabelle Alamome, Dominique Drapier, Wissam El Hage, Renaud Jardri, Stéphane Mouchabac, Marc Rabbani, Nicolas Simon, Marie-Noëlle Vacheron, Jean-Michel Azorin

**Affiliations:** 1 Department of Psychiatry, Clermont-Ferrand University Hospital, Clermont Auvergne University, Clermont-Ferrand, France; 2 Psychiatry and Medical Psychology Department, Saint-Antoine Hospital, Paris Sorbonne University, Paris, France; 3 University Hospital Psychiatry Group, Saint-Étienne University Hospital, Saint-Étienne, France; 4 Department of Psychiatry, Polyclinic of Limoges, Limoges, France; 5 University Hospital Adult Psychiatry Group, Guillaume-Régnier Hospital, University of Rennes 1, Rennes, France; 6 UMR 1253, iBrain, Tours University, Inserm, Tours, France; 7 Lille Neuroscience & Cognition Centre, INSERM U1172, Fontan Hospital, Lille University Hospital, Lille, France; 8 Medical Affairs Department, Lundbeck SAS, Puteaux, France; 9 Department of Clinical Pharmacology, Aix Marseille University, INSERM, SESSTIM, Hospital Sainte Marguerite, CAP, Marseille, IRD France; 10 Department of Psychiatry, Hospital Sainte Anne, Paris, France; 11 Department of Psychiatry, Sainte Marguerite Hospital, Marseille, France

**Keywords:** Aripiprazole, Schizophrenia, Negative symptoms, Cognition, Addiction, Pregnancy, Clozapine

## Abstract

**Background:**

Aripiprazole is a second-generation antipsychotic, efficacious in patients with schizophrenia during acute episodes. Due to its pharmacological profile, aripiprazole may be of interest in patients with specific clinical profiles who have not been studied extensively in randomised clinical trials.

**Objectives:**

To capture experience with aripiprazole in everyday psychiatric practice using the Delphi method in order to inform decision-making on the use of aripiprazole for the treatment of patients with schizophrenia in clinical situations where robust evidence from clinical trials is lacking.

**Methods:**

The scope of the survey was defined as the management of schizophrenia in adults. A systematic literature review was performed to identify the different clinical situations in which aripiprazole has been studied, and to describe the level of clinical evidence. Clinical profiles to include in the Delphi survey were selected if there was a clear interest in terms of medical need but uncertainty over the efficacy of aripiprazole. For each clinical profile retained, five to seven specific statements were generated and included in a questionnaire. The final 41-item questionnaire was proposed to a panel of 406 French psychiatrists with experience in the treatment of schizophrenia. Panellists rated their level of agreement using a Likert scale. A second round of voting on eleven items was organised to clarify points for which a consensus was not obtained in the first round.

**Results:**

Five clinical profiles were identified in the literature review (persistent negative symptoms, pregnancy, cognitive dysfunction, addictive comorbidity and clozapine resistance). Sixty-two psychiatrists participated in the first round of the Delphi survey and 33 in the second round. A consensus was obtained for 11 out of 41 items in the first round and for 9/11 items in the second round. According to the panellists’ clinical experience, aripiprazole can be used as maintenance treatment for pregnant women, is relevant to preserve cognitive function and can be considered an option in patients with a comorbid addictive disorder or with persistent negative symptoms.

**Conclusion:**

These findings may help physicians in choosing relevant ways to use aripiprazole and highlight areas where more research is needed to widen the evidence base.

**Supplementary Information:**

The online version contains supplementary material available at 10.1186/s12888-022-04008-9.

## Introduction

Second-generation antipsychotic drugs (SGAs) were introduced over twenty years ago for the treatment of schizophrenia. These agents are now generally considered to be the preferred treatments for the management of schizophrenia due to their lower propensity to cause extrapyramidal side-effects compared to first-generation antipsychotics (FGAs), such as haloperidol [1]. Aripiprazole is an SGA that is relatively selective for the D_2_ dopamine receptor, at which it acts as a partial agonist [2–4]. It has also been suggested that aripiprazole may selectively interact with dopamine pathways involved in salience attribution and in psychotic manifestations rather than in motor function [5], which may help explain its clinical profile. This selectivity may result from the ability of partial agonists such as aripiprazole to stabilise a different molecular conformation of the D2 receptor upon binding compared to full agonists or antagonists. As a consequence, a particular subset of downstream signalling pathways is selectively activated [6, 7]. This concept has been termed ‘biased agonism’ and may be a fertile approach for future innovative drug development [8, 9].

The original pharmacological profile of aripiprazole may have consequences for its clinical profile. For instance, there is also some evidence that aripiprazole improves quality of life to a greater extent than certain SGAs [10]. Importantly, the adverse event profile of aripiprazole is different from that of some of the mixed dopamine-serotonin antagonists [11–13]. In addition, it has been postulated that it may be of particular use in certain specific clinical domains, such as in patients with persistent negative symptoms, comorbid substance abuse or in partial responders to other antipsychotic drugs [14, 15]. Although the utility of aripiprazole in some of these situations has been well established from randomised clinical trials [13], in others high-level evidence is missing, and existing practice guidelines provide no guidance. Moreover, the overall profile of the clinical domains in which aripiprazole is used in everyday clinical practice remains unclear [14]. Evidence-based medicine, however, integrates the most robust published evidence with individual clinical experience and patient preferences [16], and drawing on the experience of psychiatrists who use aripiprazole in their everyday clinical practice may provide useful guidance on how this medication may best be used.

The objective of this study was to capture experience with aripiprazole in psychiatric practice in order to shed light on some of these less well-documented aspects of the clinical profile of aripiprazole. To this end, we have performed a Delphi survey of practicing psychiatrists in France based on a targeted literature review.

## Methods

In order to address the appropriateness of the use of aripiprazole in different clinical domains, we performed a Delphi survey of hospital-based psychiatrists in France. The implementation of the study was overseen by a scientific committee of eleven psychiatrists (the authors), who designed the Delphi questionnaire and interpreted the findings. These experts were all hospital-based, and were selected due to their experience in the field of schizophrenia and antipsychotics. The study was sponsored by Lundbeck France and Otsuka Pharmaceutical France, who performed the targeted literature review.

The study involved three consecutive and complementary approaches. In the first step, a review of the literature was performed by the study sponsors in order to identify the different clinical domains in which aripiprazole has been studied in adult patients with schizophrenia, and the strength of the clinical evidence was assessed.

In a second step, the results of the literature review were circulated to all members of the scientific committee, who met to choose the clinical domains to be presented to the Delphi panel. These domains were selected on the basis of the level of clinical evidence, their own clinical experience and the medical needs using a Metaplan® approach. This is a visualisation technique for facilitating decision-making in group discussions [17]. For the agreed domains, the scientific committee formulated specific questions relating to how aripiprazole is used in everyday clinical practice to be presented to participants in the Delphi survey.

The third step consisted of the Delphi survey itself, which was performed between October 2020 and March 2021. Participants were recruited from all sectors of the French healthcare system (public and private; general hospital or psychiatric hospital), with the exception of full-time community-based psychiatrists who were excluded as they typically do not treat patients with schizophrenia. The scope of the survey was defined as the suitability of aripiprazole treatment in a selection of specific clinical domains in the management of schizophrenia in adults.

The Delphi survey method is a procedure for building an expert consensus originally developed by the RAND Corporation in the 1950s [18, 19], widely used in medical research for building a consensus based on expert opinion and practice where high-level evidence from interventional studies is unavailable [20, 21]. A panel of experts is recruited and asked to indicate their level of agreement with a range of propositions. In the most frequent application of the method, this is done using a visual analogue, numerical or Likert scale. The process is iterative, with the propositions being adapted between rounds in order to facilitate achieving a consensus. Panellists never meet and complete the survey individually, which limits the possibility of concertation or herd effects. The development of web-based surveys over the last twenty years has facilitated the implementation and use of Delphi surveys. In the field of mental health, the Delphi method has been frequently applied to pragmatic research questions that may not be addressed specifically by randomised clinical trials [22–25].

### Study participants

Potential panellists were identified from professional listings of psychiatrists in France working in public or private hospitals or clinics. Psychiatrists working full-time in the community were not eligible. Panellists did not receive remuneration for their participation. We used an iterative random selection process, adjusted by *département* (French administrative geographical division of the country). We initially fixed a target of 60–70 participants, in order to ensure that around thirty completed both rounds of the survey, which is considered an appropriate number to optimise the reliability of the output of the group [18]. We then selected 400 psychiatrists at random from the listing, weighted by the total number of psychiatrists in each *département*, who were contacted by e-mail. This process was repeated with additional batches of psychiatrists at two-weekly intervals until the target number of 60–70 participants was achieved at which time the survey was closed. A reminder e-mail was sent two weeks after the original contact. A total of four batches of invitations was required. The participant invitation lists were drawn up and managed by the study moderator and the identities of the participants were not communicated to the scientific committee or to the study sponsor.

### Literature search

In order to identify potential items for the questionnaire, a systematic literature search was performed in the Medline database using the PRISMA methodology [26]. The goal of this search was to identify clinical situations in which the use of aripiprazole in schizophrenia in adults had been evaluated. All formulations of aripiprazole (oral tablets, intramuscular injectable and depot formulations) were considered in the literature review. The search terms used are listed in Table 1 and a PRISMA flow diagram of the selection process is provided in the Supplementary Information (Additional Fig. 1). Titles and abstracts of the identified publications were screened for relevance by two independent reviewers. Full texts of retained publications were individually reviewed to assess eligibility and the selection validated by an independent reviewer. References of retained publications were checked manually to ensure that no relevant publications had been missed. Any disagreements were discussed and, where possible, resolved by consensus. In a next step, the retained articles were classified by clinical domain and were ranked by evidence grade according to the classification system used by the French health authorities [27] (provided in the Supplementary Information: Additional Table 1). The retained publications were grouped into fourteen clinical domains.

**Table 1:** Literature search strategy

Database searched	Medline
Key words Search terms	Aripiprazole, Abilify, OPC 14,597 and schizophrenia (("aripiprazole"[Title/Abstract]) OR ("abilify"[Title/Abstract]) OR ("opc 14,597"[Title/Abstract])) AND ((schizophren*[Title/Abstract]) OR ("schizophrenia"[MeSH Major Topic])) Completed in a second step by:(("aripiprazole"[Title/Abstract]) OR ("abilify"[Title/Abstract]) OR ("opc 14,597"[Title/Abstract])) AND (pregnan* OR fertility OR lactation OR breastfeeding)
Date filters (Medline)	Up to May 2020
Language filters (Medline)	English, French
Other filters (Medline)	Species (human) Age (Adult: > 18 years

### Selection of themes and development of the questionnaire

For each of the fourteen clinical domains, the scientific committee voted on how appropriate the use of aripiprazole was, on the basis of the strength of the available evidence and their own clinical experience. For each domain, each member of the committee chose between situations in which the use of aripiprazole is clearly appropriate, those in which it is clearly not appropriate, and those in which there is a doubt. The scientific committee then prioritized the domains for which the majority of members considered that, in terms of the published evidence, there was a doubt concerning the utility of aripiprazole but where there was a clear interest in terms of medical need. Five domains were selected as being related to a significant unmet clinical need and thus the most important to include in the questionnaire (Table 2). The number and type of publications identified in the literature review which are relevant to these five themes are listed in the Supplementary Information (Additional Table 2).

**Table 2:** Themes evaluated and retained for the Delphi survey

	Clinical domain for aripiprazole prescription:
1	First episode psychosis
2	Acute exacerbation or relapse of psychosis
3	Agitation (intramuscular injection)
4	Long-term maintenance therapy
5	Onset of cardiovascular or metabolic disorders
6	Onset of hyperprolactinemia or sexual disorders
**7**	**Occurrence of cognitive disorders**
**8**	**Secondary negative symptoms**
9	Partial response (other than resistant schizophrenia)
**10**	**Addictive comorbidity**
**11**	**Resistant schizophrenia in association with clozapine**
12	Resistant schizophrenia as monotherapy
13	Resistant schizophrenia in association (without clozapine)
**14**	**Pregnancy**

Themes in **bold** were those retained for the Delphi survey

Finally, for each of the five retained clinical domains, between five and seven specific questions were generated using the Metaplan® method. Each question was formulated in the following way: ‘*According to your own clinical experience in the treatment of an adult patient with schizophrenia, aripiprazole is an appropriate treatment option for…*’. The final questionnaire contained 28 questions, some of which had subsidiary questions (41 items in all).

### Implementation of the survey

The Delphi questionnaires were administered and the data collected by an independent moderator, who was the only person who knew the identity of the panellists and had access to the individual votes. The moderator used a web-based survey software (*Survey Monkey*®) to invite psychiatrists to participate, to deliver the questionnaire and to collect and compile the ratings.

The study questionnaire opened with a series of screening questions to ensure that the panellist had the required experience. In order to proceed with the survey, panellists were required to confirm that they were a psychiatrist treating adult patients with schizophrenia, prescribing atypical antipsychotics, working in the hospital sector, and seeing patients in at least three of the five clinical domains of interest in the survey. The questionnaire opened with a series of questions collecting background information on the panellist (age, gender, geographical region, place and size of practice, and involvement in teaching or research activities). This was followed by the 28 questions about aripiprazole formulated as a statement for which panellists rated their level of agreement on a numerical scale from one to nine. An option of ‘no experience in this domain’ was also available, but this was never used. A free-text box was available for participants to comment on their response or on the question, if they so wished. It was not possible to skip questions. Participants could close the survey at any time and return to it when they wished.

A score of 1 – 3 was considered to indicate disagreement, a score of 7 – 9 agreement and a score of 4 – 6 undecided. Replies to the questionnaire were compiled and the proportion of panellists in agreement (or disagreement) with each statement was calculated. There is no established threshold for identifying consensus in Delphi surveys although agreement of 80% of participants is widely used [28]. However, in the present study, a low overall level of consensus was anticipated because the questions chosen were those for which the scientific committee considered that there was no clear answer; for this reason, a less stringent threshold of 65% agreement was applied. The statements reaching consensus using this criterion were identified and retained. For statements for which 50% – 65% of panellists were in agreement, the statements were rephrased for the second round.

In the second round, eleven reformulated statements were proposed to the panellists, who were asked whether they agreed or disagreed with the statement (binary choice). Replies were compiled and the proportion of panellists in agreement was calculated. Statements with which ≥ 65% of panellists agreed were considered consensual.

## Results

### Participants

In the first round, 406 psychiatrists who were sent the invitation opened the electronic mail, 84 of whom (20.7%) entered the *Survey Monkey*® interface. Of these, five refused to participate and a further seventeen either did not fulfil the eligibility criteria or did not reply to any of the questions about aripiprazole. Of the remaining 62 psychiatrists starting the questionnaire, 52 completed it (83.9%). For the second round, an invitation was sent to the 62 panellists who started the questionnaire during the first round. Of these, 49 opened the mail, 34 entered the survey interface and 33 completed the questionnaire.

The characteristics of the participants are presented in Table 3. Panellists were most frequently under 40 years old, with nine aged ≥ 60 years. The majority were men. Thirty-two had a teaching activity and sixteen were involved in research.

**Table 3:** Characteristics of Delphi participants

**Age**	*N* = 62
40 years	26 (41.9%)
40 – 49 years	13 (21.0%)
50 – 59 years	14 (22.6%)
≥ 60 years	9 (14.5%)
**Gender**	*N* = 62
Men (n, %)	38 (61.3%)
**Type of practice (principal)**	*N* = 62
University hospital	20 (32.3%)
General hospital	15 (24.2%)
Mental health hospital	18 (29.0%)
Community mental health centre	9 (14.5%)
**Number of patients with schizophrenia followed (per month)**	*N* = 62
10	7 (11.3%)
10 – 50	38 (61.3%)
50	17 (27.4%)
**Type of patients followed**	*N* = 62
Schizophrenia associated with negative symptoms	58 (93.5%)
Schizophrenia during pregnancy	40 (64.5%)
Schizophrenia associated with cognitive symptoms	58 (93.5%)
Schizophrenia associated with addictive comorbidity	57 (91.9%)
Resistant schizophrenia	58 (93.5%)
**Related activities**	*N* = 62
Teaching activity related to schizophrenia	32 (51.6%)
Research activity related to schizophrenia	16 (25.8%)

### Persistent negative symptoms

In the first round, five questions were put to the panel corresponding to different presentations of negative symptoms (Additional Table 3). Each question had two sub-questions, concerning switching of antipsychotic medication to aripiprazole on the one hand and augmentation therapy with aripiprazole on the other. Three questions obtained a consensus with respect to switching and one with respect to augmentation (Table 4). In the second round, two questions were presented (Additional Table 3), both of which achieved consensus (Table 4). Examples of verbatim obtained for this theme are provided in Additional Table 4.

**Table 4:** Consensus items from the Delphi survey

DOMAIN 1. Persistent negative symptoms	
Round 1 Q1	In the management of an adult patient with schizophrenia presenting with blunted affect associated with **residual psychotic symptoms** unimproved by current SGA treatment, a switch from the current SGA to aripiprazole is a relevant treatment option
Round 1 Q3	In the management of an adult patient with schizophrenia presenting with blunted affect associated with **psychomotor retardation** unimproved by current SGA treatment, a switch from the current SGA to aripiprazole is a relevant treatment option:
Round 1 Q4	In the management of an adult patient with schizophrenia presenting with blunted affect associated with **anxiety** unimproved by current SGA treatment, aripiprazole in association with the current SGA is NOT a relevant treatment option:
Round 1 Q5	In the management of an adult patient with schizophrenia presenting with blunted affect associated with **social withdrawal** unimproved by current SGA treatment, a switch from the current SGA to aripiprazole is a relevant treatment option:
Round 1 Q1	In the management of an adult patient with schizophrenia presenting with blunted affect associated with **other negative symptoms** partially responsive to the current SGA, addition of aripiprazole in combination with the antipsychotic is NOT a more relevant treatment option than aripiprazole in monotherapy
Round 1 Q2	In the management of an adult patient with schizophrenia presenting with blunted affect associated with **depressed mood** partially responsive to the current SGA, aripiprazole is a relevant treatment after the failure of combining the current SGA with an antidepressant
DOMAIN 2. Pregnancy	
Round 1 Q3a	Maintenance of oral aripiprazole is an appropriate option in a well-controlled adult patient who discovers her pregnancy during the first trimester
Round 1 Q5	Maintenance of oral aripiprazole is an appropriate option in an adult patient throughout the duration of her pregnancy right up to delivery
Round 2 Q1a	In an adult patient well-controlled on oral aripiprazole who finds herself pregnant, aripiprazole treatment should be maintained
Round 2 Q1b	In an adult patient well-controlled on oral aripiprazole who is planning a pregnancy, aripiprazole treatment should be maintained
Round 2 Q2b	In an adult patient well-controlled on depot aripiprazole who is planning a pregnancy, aripiprazole treatment does not need to be switched to the oral formulation of aripiprazole
**DOMAIN 3: COGNITIVE DYSFUNCTION**	
Round 1 Q1	In the management of an adult patient with schizophrenia, aripiprazole is a relevant treatment option to preserve cognitive function
Round 1 Q6	In the management of an adult patient with schizophrenia presenting with cognitive problems appearing during treatment with the current antipsychotic, a switch to aripiprazole is a relevant treatment option if the patient is treated with an FGA
Round 2 Q2	In the management of an adult patient with schizophrenia presenting with cognitive problems appearing during treatment with the current antipsychotic, a switch to aripiprazole is a relevant treatment option
**DOMAIN 4: ADDICTIVE COMORBIDITY**	
Round 1 Q1	Aripiprazole is an appropriate option in an adult with schizophrenia and a comorbid addictive disorder
Round 1 Q5	Aripiprazole is an appropriate option for treatment of a first psychotic episode in a patient with a comorbid cannabis use disorder
Round 2 Q1	In an adult patient well-controlled on another antipsychotic who has an unresolved addictive disorder, add-on aripiprazole treatment is NOT an appropriate option
Round 2 Q2a	In an adult patient not controlled on another antipsychotic who has an unresolved addictive disorder, switching to aripiprazole treatment is a relevant therapeutic alternative
Round 1 Q2	In the management of an adult patient with schizophrenia with clozapine resistance, aripiprazole is a relevant treatment option if negative symptoms persist
Round 2 Q1	In the management of an adult patient with schizophrenia presenting a partial response to clozapine, aripiprazole is a relevant treatment option

### Pregnancy

In the first round, six questions were put to the panel (Additional Table 3). Two of these received a consensus (Table 4). In the second round, four questions were presented (Additional Table 3), for three of which a consensus was obtained (Table 4). Examples of verbatim obtained for this theme are provided in Additional Table 4.

### Cognitive dysfunction

In the first round, seven questions, some with sub-questions, relating to cognitive symptoms were put to the panel (Additional Table 3). Two questions obtained a consensus in this round (Table 4). In the second round, a further two questions were presented (Additional Table 3), one of which achieved consensus (Table 4). Examples of verbatim obtained for this theme are provided in Additional Table 3.

### Addictive comorbidity

In the first round, seven questions were put to the panel (Additional Table 3). Two of these received a consensus (Table 4). In the second round, two questions were presented (Additional Table 3), both of which achieved consensus (Table 4). Examples of verbatim obtained for this theme are provided in Additional Table 4.

### Augmentation therapy for clozapine resistance

In the first round, four questions were put to the panel relating to patients resistant to clozapine therapy (Additional Table 3). Certain questions had two or three sub-questions. One question obtained a consensus (Table 4). In the second round, a single question was presented (Additional Table 3), which achieved consensus (Table 4). Examples of verbatim obtained for this theme are provided in Additional Table 4.

## Discussion

The Delphi survey identified a relatively limited number of consensus statements on the themes of interest in the study. These consensus points nevertheless identify a number of clinical domains where, in spite of the limited published data available, participants feel, based on their own clinical experience, comfortable about the use of aripiprazole. These are reviewed by theme hereunder.

### Persistent negative symptoms

Negative symptomatology constitutes an important dimension of schizophrenia and its adequate management remains an important unmet medical need in the treatment of schizophrenia [29–32]. Patients with pronounced negative symptoms tend to be less functional and have poorer outcomes than those in whom such symptoms are milder [11, 33–37]. It has been clear for several decades that negative symptoms respond less well to antipsychotic medication than acute psychotic symptoms. Over forty years ago, it was recognised that FGAs did not markedly improve negative symptoms, and could even potentially aggravate these symptoms [38, 39]. These observations contributed to the search for antipsychotics with a broader pharmacological profile and to the development of SGAs. However, the clinical benefit of these SGAs in patients with persistent negative symptoms has been a matter of debate [40–43]. Indeed, recent epidemiological findings suggest that around one-half of otherwise well-controlled patients taking an established antipsychotic treatment present with persistent residual negative symptoms [34]. As a partial agonist, aripiprazole is of interest for treating both the positive and negative dimensions of the disorder.

The Delphi panellists achieved consensus that aripiprazole is an appropriate treatment option in patients with persistent negative symptoms. For treating patients with blunted affect, a dopamine receptor partial agonist was considered a promising choice. In general, panellists thought that it would be more appropriate to treat patients presenting with persistent negative symptoms with an antipsychotic in monotherapy than in combination. Consequently, in a patient with residual negative symptoms on the current antipsychotic, a switch to aripiprazole was preferred to augmentation therapy. There is no information in the literature to support the use of augmentation with aripiprazole except in the context of clozapine resistance [44] (see below). With respect to switching to monotherapy with aripiprazole, only one comparative prospective study has been published, but this is very poorly documented; it reported improvements in the negative subscale of the Positive and Negative Syndrome Scale (PANSS) in patients treated with aripiprazole [45]. On the other hand, several case reports have been published that describe benefits on negative symptoms following a switch to aripiprazole [46–48]. Several panellists also stated that they would initially attempt to manage depressive or anxious symptoms by prescribing an antidepressant or anxiolytic rather than by changing antipsychotic medication. There is no information in the literature concerning the sequencing of antidepressant/anxiolytic treatment and aripiprazole in the literature (Table 5).

**Table 5:** Opinion of the scientific steering committee – persistent negative symptoms

• A switch to aripiprazole should be considered in patients treated with an SGA in case of: – blunted affect associated with residual psychotic symptoms – psychomotor retardation – social withdrawal
• In patients otherwise well-controlled on their current antipsychotic medication with depressed mood or anxiety, specific therapies for these symptoms should be tried before considering changing antipsychotic medication
• Aripiprazole should be considered as an appropriate choice, in combination with an antidepressant, for patients with blunted affect and depressed mood

### Pregnancy

Pregnancy and the peripartum period present a number of risks to women with schizophrenia treated with antipsychotic medication. Discontinuing treatment may result in re-emergence of psychotic episodes which may endanger the mother or baby (or both), whereas continuing treatment may expose mother and baby to drug-related adverse events. In addition, compared to the general population, women with schizophrenia more frequently present pregnancy risk factors, such as smoking during pregnancy, poor nutritional status, alcohol or recreational drug use, and high body mass index [49–51]. In addition, women with schizophrenia may have poor knowledge of contraceptive methods and little involvement in contraceptive decisions, and for this reason, be more likely to have unplanned or unwanted pregnancies [52–54]. For most stable patients, well-controlled on their medication, the discontinuation of treatment in women treated for schizophrenia, before or during pregnancy, is associated with a high risk of a new psychotic episode, notably in the case of abrupt termination of treatment [55]. The presence of maternal psychotic symptoms at birth is likely to interfere with the mother’s ability to care for the new-born child and may be detrimental to establishing a healthy bond between mother and infant [56–59]. Estimating the risks associated with use of SGAs during pregnancy is difficult, since pregnant women are excluded from randomised clinical trials and no large prospective pregnancy registries have been initiated in schizophrenia. Certain SGAs have metabolic and cardiovascular effects that may be of relevance in pregnancy, including gestational diabetes [60–63] and perinatal cardiac rhythm disturbances [64]. Given the limited amount of evidence, it is difficult to appreciate whether SGAs carry any teratogenic risk in humans, although, if it exists, this risk is likely to be low [50, 65–69]. An increased risk of congenital malformations has been reported in women exposed to risperidone or olanzapine [70, 71]. Whether exposure to antipsychotics in utero exposes the child to any neurodevelopmental toxicity is unknown [72], although there is no evidence for any deleterious psychiatric outcome [73].

Delphi participants reached a consensus that treatment with aripiprazole could and should be maintained during pregnancy or in a patient who finds herself pregnant, as long as psychiatric symptoms are adequately controlled. This is supported by the limited amount of published data available. Recent data from the National Pregnancy Registry for Atypical Antipsychotics in the USA found no elevated risk of major malformations in infants born to mothers exposed to aripiprazole during pregnancy [74], and this was also the conclusion of a number of smaller cohort studies [71, 75, 76]. A small number of case reports of aripiprazole exposure during pregnancy or lactation have been described, which have involved different aripiprazole doses, different lengths of treatment, and different timings of prenatal exposure [77–85]. Most of these have described uneventful pregnancies resulting in a healthy baby [77–80, 82, 84, 85]. Nonetheless, there have been reports of foetal tachycardia [81] and transient breathing difficulties [83] in infants exposed to aripiprazole in utero during late pregnancy. Small case series of pregnancies exposed to aripiprazole have also reported a slightly shorter gestation and lower birth weight, and a mildly elevated risk of gestational hypertension [86], as well as a possible signal of miscarriage [87]. Nonetheless, attribution of causality in these cases was not clear. There was no agreement between participants as to the utility of switching to aripiprazole in a patient well-controlled on another antipsychotic or on switching between depot and oral formulations of aripiprazole. Although a majority of panellists agreed that treatment with aripiprazole could be continued right up to delivery, some felt that it should be discontinued in the last month if the clinical state of the patient allowed. With respect to breastfeeding, transfer of aripiprazole has been demonstrated across the placenta [83, 85, 88] and also into breast milk [79, 89], so there is some potential for harmful effects on the foetus or the new-born (Table 6).

**Table 6:** Opinion of the scientific steering committee—pregnancy

• As a general principle, exposure to pharmaceutical products during pregnancy should be kept to the strict minimum necessary
• Aripiprazole should only be initiated or maintained in women planning a pregnancy, or who find themselves pregnant, after a careful evaluation of the benefits and risks of treatment
• This evaluation should be multidisciplinary, including the psychiatrist, obstetrician, midwife, general practitioner, and involving the patient herself and any carers or peer support persons
• Treatment during pregnancy may be envisaged if the patient is well-controlled with aripiprazole, or has responded to this antipsychotic during a previous episode, or if there is an important identified risk of side effects with alternative treatment options
• In women who are currently well-controlled on an appropriate dose of aripiprazole and who are planning a pregnancy or find themselves pregnant, and in the absence of any identified risks, treatment can be continued
• In patients treated with the depot formulation of aripiprazole, a switch to the oral formulation is not generally necessary
• In a well-controlled patient, dose adjustment is not generally justified
• With appropriate monitoring, treatment can be continued right up to delivery
• The new-born infant should be assessed carefully for any residual drug effects after delivery
• Given the long elimination half-life of aripiprazole (75 to 146 h), there is a risk of accumulation in breast-fed infants; for this reason, breast-feeding with aripiprazole is not recommended

### Cognitive dysfunction

Cognitive dysfunction is a fundamental aspect of schizophrenia, distinct from the positive and negative symptom domains [90, 91]. The severity of cognitive impairment is believed to be strongly associated with functional outcome and the ability to live an independent life [92, 93]. Effective management of cognitive dysfunction in schizophrenia is thus a crucial challenge for improving the long-term outcome of therapy. First-generation antipsychotics were perceived as having, at best, minimal effects on cognitive performance, and possibly even impairing performance [94, 95]. The introduction of the SGAs raised hopes that they would have a more incisive effect on cognitive function than the earlier generation of treatments [94, 96]. However, 25 years later, the real impact of SGAs on cognitive function remains a matter of debate [97–99]. In addition, it is probably simplistic to envisage SGAs as a homogeneous class, and there may be significant differences between drugs in terms of their response profile. In this respect, aripiprazole, which facilitates prefrontal dopaminergic neurotransmission [100], may be of interest to improve cognitive performance in schizophrenia.

The Delphi panellists supported the view that aripiprazole was a relevant treatment option for patients with schizophrenia presenting with cognitive dysfunction. In particular, they agreed that a switch to aripiprazole would be appropriate in patients with persistent cognitive dysfunction on their current antipsychotic medication. This opinion is consistent with the limited published data. The targeted literature search identified four studies that had evaluated the effects of aripiprazole on cognitive symptoms of schizophrenia and that could be graded as Evidence Level 2 [101–104]. All of these studies, which used a variety of paradigms, reported beneficial effects of aripiprazole on certain cognitive domains and some evidence of superiority with respect to risperidone or olanzapine. One of these studies reported benefits in the context of switching from an FGA to aripiprazole [103], and a second benefit following adjunctive treatment with aripiprazole in patients with persistent cognitive dysfunction on monotherapy with risperidone or olanzapine. However, patterns of improvement across cognitive domains were not consistent within or between studies, which makes it difficult to draw an unequivocal conclusion from them. In addition to these studies, a number of prospective or retrospective case series have been reported [47, 105–109], which all reported benefits on cognitive function of switching to aripiprazole. Panellists emphasised the importance of non-pharmacological approaches in addition to antipsychotic medication, and in particular cognitive remediation, for which the evidence base for efficacy is consistent [110]. This view is supported by findings from a prospective comparative study which evaluated the impact of combining cognitive remediation with aripiprazole or risperidone and reported a possible synergistic effect for aripiprazole [111] (Table 7).

**Table 7:** Opinion of the scientific steering committee – cognitive function

• A switch to aripiprazole should be considered in patients in whom cognitive symptoms appear under treatment with an FGA
• If preservation of cognition function is an issue, aripiprazole would be an appropriate choice of SGA
• To preserve cognitive function, the minimum effective dose of aripiprazole should be identified and used
• In the management of an adult with schizophrenia currently treated with aripiprazole but presenting with residual cognitive symptoms, a dose reduction strategy can be considered
• To preserve cognitive function, aripiprazole monotherapy should be preferred to aripiprazole in combination therapy whenever possible
• Psychosocial interventions, such as cognitive remediation, are recommended together with antipsychotic medication to improve cognition
• In patients otherwise well-controlled on their current antipsychotic medication but presenting with persistent cognitive symptoms, combination with aripiprazole could be considered

### Addictive comorbidity

The prevalence of comorbid addictive disorders in patients with schizophrenia is high compared to the general population, and many of these patients present with multiple addictions [112]. These patients are a particular challenge for the treating psychiatrist, as they may have poorer treatment persistence [113] and compliance [114], poorer symptom control [115] or more pronounced extrapyramidal symptoms [116], poorer prognosis [114, 117], higher mortality [118] and poorer social integration [115] than patients without addictive comorbidity. Treatment of patients with schizophrenia with FGAs may aggravate addictive comorbidity and compromise attempts to stop or reduce substance use [119, 120]. In contrast, there is evidence that certain SGAs may be beneficial in these patients [121] and possibly reduce craving and facilitate a reduction in substance use [122]. In this respect, aripiprazole is a particularly interesting candidate due to its partial agonist profile, which may allow a preserved hedonic response.

There was a consensus among the Delphi panellists that aripiprazole had a place in the management of patients with schizophrenia and a comorbid addictive disorder. However, they thought that it was difficult to generalise experiences and opinions across the many different types of addictive behaviour encountered in patients with schizophrenia. Certain panellists considered that the partial agonist properties of aripiprazole and its ‘pro-cognitive’ effects made it a promising candidate for the treatment of this type of patient. In everyday practice, panellists commented that, although aripiprazole treatment did reduce craving in some patients, this was not a consistent finding. Published randomised comparative clinical trials, on the other hand, have generally reported significant reductions in craving in patients treated with aripiprazole, for smokers [123], cocaine users [124] and cannabis users [125]. Similarly, uncontrolled cohort studies and case series or reports have also reported reductions in craving for various addictive substances following aripiprazole treatment [124, 126, 127]. In patients with a first psychotic episode and, as long as a diagnosis of schizophrenia could be made with confidence, panellists often prescribed aripiprazole to patients with a substance use disorder, particularly cannabis, in the hope that this medication would bring psychotic symptoms under control and help reduce substance abuse. There is very little published data on aripiprazole and cannabis addiction and the panellists’ experience is thus particularly valuable. A randomised naturalistic study comparing depot formulations of aripiprazole and paliperidone reported significantly greater reductions in craving in patients using cannabis following one years’ treatment with aripiprazole compared to paliperidone [125]. The only other published study that evaluated aripiprazole in this context was a retrospective case series in 17 cannabis users which reported no change in scores on the Severity of Dependence Scale after six months of treatment with the depot formulation [128]. There was a consensus that, in patients with an unresolved addictive disorder on their current antipsychotic, adjunctive treatment with aripiprazole in order to treat the addiction would not be an appropriate solution. If a patient’s symptoms were well-controlled on the current antipsychotic, then it may not always be appropriate to switch to aripiprazole just in the hope of having some impact on the addictive comorbidity. In contrast, if the patient was not well controlled and a switch of antipsychotic treatment was planned, then aripiprazole may be a suitable choice in this patient group. No clinical studies have yet addressed the effect of aripiprazole in different switching strategies in patients with addictive comorbidities under treatment with another antipsychotic. It was also stressed by several participants that the prescription of aripiprazole to a patient with an addictive comorbidity should not obviate the importance of providing a specific and appropriate treatment for the addictive disorder itself (Table 8).

**Table 8:** Opinion of the scientific steering committee – addictive comorbidity

• In general, aripiprazole is a relevant choice for the treatment of patients with schizophrenia and an addictive comorbidity
• Aripiprazole should be considered as an appropriate choice for patients requiring a first-line antipsychotic treatment for a first psychotic episode who have a cannabis use disorder
• Aripiprazole should be considered as an appropriate therapeutic alternative for patients with an addictive comorbidity who are poorly controlled on their current antipsychotic
• In contrast, the presence of an addictive comorbidity is not a sufficient reason to switch a patient who is well-controlled on current medication to aripiprazole
• Management of patients with schizophrenia and an addictive comorbidity should involve both antipsychotic treatment, with for example aripiprazole, and therapies targeted specifically at reducing substance misuse

### Augmentation therapy for clozapine resistance

Perhaps up to half of patients with treatment-resistant schizophrenia (TRS) do not respond satisfactorily to clozapine [129] and these patients often have particularly severe disease [130]. Although a number of strategies have been proposed for treating patients with TRS who do not respond to clozapine, including augmentation with other antipsychotics, there is no consensus on which strategy represents the most beneficial approach [131–133]. Augmentation with aripiprazole may be of interest in this context.

Clozapine resistance is a complex issue and the treating physician's judgment may differ from that used in clinical studies. Resistance does not only relate to persistence of psychotic symptoms, but also to poor global functioning, persistent lack of initiative and involvement, as well as reduced social skills. These symptoms are the consequence of poor cognitive function, poor social involvement, poor reward capacities and lack of environmental stimuli. For the clinician, treatment decision-making in these cases is not limited to observation of insufficient improvement of measurable symptoms, but also to difficulty in restoring patient motivation and autonomy. In this respect, the choice of intervention in clozapine resistance may be driven by attitudes to resistance that are not easily quantified, and are based on a more pragmatic approach. This difference between outcomes used in clinical trials and physician judgement in real-world practice illustrates the interest of the Delphi method for guiding clinical practice.

Panellists considered that, if augmentation of clozapine with another SGA was being considered, then aripiprazole would be an attractive choice, since it has a complementary mechanism of action to clozapine. In addition, compared to certain other SGAs, aripiprazole carries a lower risk of metabolic side-effects [12], and is not likely to have a synergistic effect with clozapine on weight gain, dyslipidaemias and hyperglycaemia. Two systematic reviews have evaluated the interest of antipsychotic augmentation strategies in clozapine-resistant schizophrenia [129, 134], one conducted under the aegis of the Cochrane collaboration [135]. Both noted the limited number and poor quality of the available studies. The studies reviewed provided inconsistent information on the benefits of augmentation with aripiprazole. The Cochrane review identified one study demonstrating superiority of aripiprazole augmentation over quetiapine augmentation [136], and one study which reported no benefit [137]. In the second systematic review, aripiprazole and penfluridol, were the only antipsychotics included in the meta-analysis to show a significant reduction in total symptom score compared to placebo, and the effect size was highest for aripiprazole. Panellists expected benefits of augmentation therapy particularly in case of residual negative symptoms. This notion is supported by the findings of a double-blind, randomised and placebo-controlled clinical trial of aripiprazole augmentation in clozapine-resistant schizophrenia [138], which concluded that aripiprazole augmentation did not improve total symptom severity, but may provide a benefit in the negative symptom domain. Panellists thought that, in case of residual depressive symptoms, addition of an antidepressant may be more appropriate.

It was considered that there was insufficient information available to position aripiprazole with respect to ECT in this context. Panellists were sceptical that augmentation with aripiprazole would lead to a reduction in the adverse events of clozapine unless it was possible in parallel to decrease the dose of clozapine. Several participants emphasised the importance of monitoring plasma concentrations of clozapine prior to starting augmentation therapy to ensure that plasma levels are adequate, and to adjust the dose if necessary. This is particularly important in heavy smokers, since smoking strongly induces CYP1A2, the enzyme that metabolises clozapine [139]. Concerning the interest of a depot formulation of aripiprazole, panellists thought that this would principally be appropriate in patients whose observance was poor (Table 9).

**Table 9:** Opinion of the scientific steering committee – clozapine resistance

• Augmentation with aripiprazole should be considered in patients with TRS treated with clozapine in case of persistence of symptoms; this is of interest when negative symptoms persist
• Augmentation with aripiprazole should be attempted even in patients who did not respond to aripiprazole monotherapy prior to clozapine treatment

### Methodological issues

The aim of the recruitment process was to ensure participation of > 30 participants in both rounds of the survey. This number has been proposed by the developers of the Delphi method as being optimal for minimising the risk of an erroneous outcome and for generating a reliable consensus [18]. However, in practice, the number of participants in Delphi surveys is frequently lower: a systematic review of eighty Delphi studies in the field of healthcare published in 2011 reported that the median number of participants was seventeen [140]. We anticipated that certain potential participants may not be eligible or not prepared to complete the survey. In addition, it was expected that not all participants in the first round would be prepared to participate in the second round. In another recent Delphi survey in France which also recruited patients from professional listings, only two-thirds of first-round participants took part in the second round [141]. From these considerations, we targeted 60–70 physicians starting the survey. Once this number was reached, the survey was closed. In fact, 84 psychiatrists opened the survey, of whom seventeen did not fulfil the eligibility criteria (for example, they did not prescribe antipsychotic medication) and five declined to participate. Of the 62 psychiatrists who started the questionnaire, all but ten completed it. The relatively small number of psychiatrists who declined to participate or failed to complete the questionnaire suggests that participants who entered the survey found the content interesting and relevant. As anticipated, a proportion of participants in the first round did not take part in the second round, and this proportion was similar to that reported in the recent gastroenterology Delphi survey in France [141]. The final number of psychiatrists who completed the survey was 33, which was compliant with the initial target of at least thirty participants.

Of the 406 psychiatrists who were contacted and who opened the mail, 84 entered the survey. This response rate (20.7%) rate is thus rather low. The main reason for this is probably that participants were not co-opted by personal contact by the scientific committee, but were recruited by ‘cold call’. The co-option approach ensures that potential participants contacted are known from the outset to be interested in the subject matter and are likely a priori to be interested in participating. In contrast, using the ‘cold-call’ approach, it is not possible to target those physicians most likely to participate. It was decided to use the ‘cold-call’ rather than the co-option approach in order to capture a wide as possible cross-section of antipsychotic prescribers in France. The psychiatrists received an unsolicited e-mail from a sender address with which they were not familiar. The sender had no information on the psychiatrist other than the hospital or clinic in which they worked, and it is likely that some of the psychiatrists to whom the invitation was sent were not involved in the treatment of patients with schizophrenia. In previous surveys in the field of mental health, participation rates are generally > 80% for those in which targeted participants are co-opted [23, 142, 143] and < 20% for those using ‘cold call’ recruitment [144–146], although in many publications the response rate was not reported. In the light of this previous experience, the response rate observed in the present study and the low number of psychiatrists who explicitly refused to participate can be considered acceptable. No financial incentive was offered to participate and the survey was internet-based. Both of these features have been shown to be to associated with lower response rates in physician surveys [147–149]. It should also be noted that, to some extent, the response rate is a consequence of the recruitment target. The survey was closed once the target of 60–70 participants had been reached. It is possible that if the survey had been kept open longer or if the mailing of reminders had been more intensive, then more of the contacted psychiatrists may have responded. Finally, the survey was implemented during the pre-vaccination COVID-19 epidemic, when hospital resources and staff were under significant pressure, and this may also have discouraged certain psychiatrists from participating.

The current study has a number of more general limitations. Firstly, the literature review was carried out on one source (Medline) and may thus be not exhaustive. Secondly, members of the scientific committee and the Delphi participants were principally hospital-based. Therefore, there might be limitations on the representativeness of the clinical experience captured which may potentially lead to difficulty in generalising the results to a wider population or a different healthcare system.

## Conclusion

This Delphi survey has shed light on current clinical practice with aripiprazole in clinical domains where the evidence base from published clinical trials is narrow. The ideas that emerge may be helpful for physicians in choosing the most promising ways to use this antipsychotic. They also highlight areas where more research is needed, either from formal clinical trials or pragmatic observational studies in everyday clinical practice, in order to widen the evidence base. Future research should also investigate patient perceptions of aripiprazole in these clinical domains.

## Supplementary Information

**Additional file 1:** Place of the partial dopamine receptor agonist aripiprazole in themanagement of schizophrenia in adults: a Delphi consensus study. **Additional Table 1.** Evidence level classification of the Frenchhealth authorities**. Additional Table 2.** Studies identified in the literature review**. Additional Table 3.** Questions asked in the Delphisurvey**. Additional Table 4.** Examples of verbatim from theDelphi survey**. Additional Figure 1.** PRISMAflow diagram for the literature search**.**

## Data Availability

Anonymised data on the results of the survey are held by the study moderator on behalf of the authors. Reasonable requests for access to this data can be addressed to the author for correspondence.
