# Supplementary material for: Place of the partial dopamine receptor agonist aripiprazole in the management of schizophrenia in adults: a Delphi consensus study
Source: BMC Psychiatry. 2022 May 28;22:364. doi: 10.1186/s12888-022-04008-9 (PMC9142729; doi:10.1186/s12888-022-04008-9)

Place of the partial dopamine receptor agonist aripiprazole in the management of schizophrenia in adults: a Delphi consensus study

**Supplementary Information**

Additional Table 1 Evidence level classification of the French health authorities

| **Evidence grade** | **Definition** |
| --- | --- |
| A. Strong | - Study design appropriate for answering the scientific question as well as possible - Study conducted without major bias - Statistical analysis appropriate to address the objectives of the study - Statistical power adequate |
| B. Intermediate | - Study design appropriate for answering the scientific question as well as possible - EITHER Statistical power insufficient (too small sample enrolled or found to be inadequate *a posteriori*) - OR minor divergence in the conduct of the study |
| C. Weak | - All other studies |
| **Recommendation grade** | **Evidence level documented in the scientific literature** |
| A. Established scientific evidence | Level 1.   - Well-powered randomised comparative clinical trials - Meta-analyses of randomised comparative clinical trials - Decision analysis based on well-conducted studies |
| B. Presumption of proof | Level 2.   - Low-powered randomised comparative clinical trials - Well-conducted non-randomised studies - Cohort studies |
| C. Low level of proof | Level 3.   - Case-control studies   Level 4.   - Comparative studies with major bias - Retrospective studies - Case series - Descriptive epidemiological studies (cross-sectional or longitudinal) |

Additional Table 2 Studies identified in the literature review

|  | **All studies** | **EVIDENCE GRADE** | | | |
| --- | --- | --- | --- | --- | --- |
|  |  | **Level 1** | **Level 2** | **Level 3** | **Level 4** |
| **Persistent negative symptoms** | 5 |  | 1   - 1 P, O |  | 4   - 1 P, O - 3 case reports/series |
| **Pregnancy** | 12 |  |  |  | 11   - 10 case reports/series - 1 case control-study - 1 qualitative systematic review |
| **Cognitive dysfunction** | 16 | 4   - 1 P, R, O - 1 O, R - 1 R, DB - 1 R, DB, PC |  |  | 12   - 1 *Post hoc* analysis - 1 P, N, C - 3 P cohort studies - 1 P, C - 1 Cross sectional study - 1 Cross-over study - 1 Imaging study - 3 case reports/series |
| **Addictive comorbidity** | 10 | 1   - 1 RC | 1   - 1 R, N |  | 8   - 1 P, R* - 1 OL, C - 6 case reports/series |
| **Augmentation therapy for clozapine resistance** | 21 | 4   - 2 MA - 2 DB, R, PC | 2   - 1 DB, R, PC - 1 R, SB |  | 15   - 15 Case series/reports |

*Although this was a prospective, randomised study, responses to individual SGAs were not individualised, and for this reason the study is classed as Evidence Level 4.

C: controlled; DB: double-blind; MA: meta-analysis; N: naturalistic; O: Observational; OL: open-label; P: prospective; PC: placebo-controlled; R: randomised, SB: single blind.

Additional Table 3 Questions asked in the Delphi survey

|  | **DOMAIN 1: PERSISTENT NEGATIVE SYMPTOMS** |  |
| --- | --- | --- |
| N° | **First round** |  |
| 1. | In the management of an adult patient with schizophrenia presenting with blunted affect associated with **residual psychotic symptoms** unimproved by current SGA treatment, aripiprazole is a relevant treatment option: |  |
|  | a) in association with the current SGA | No agreement |
|  | b) as a switch option for the current SGA | **Consensus**: agreement |
| 2. | In the management of an adult patient with schizophrenia presenting with blunted affect associated with **depressed mood** unimproved by current SGA treatment, aripiprazole is a relevant treatment option: |  |
|  | a) in association with the current SGA | No agreement |
|  | b) as a switch option for the current SGA | Trend to agreement |
| 3. | In the management of an adult patient with schizophrenia presenting with blunted affect associated with **psychomotor retardation** unimproved by current SGA treatment, aripiprazole is a relevant treatment option: |  |
|  | a) in association with the current SGA | No agreement |
|  | b) as a switch option for the current SGA | **Consensus**: agreement |
| 4. | In the management of an adult patient with schizophrenia presenting with blunted affect associated with **anxiety** unimproved by current SGA treatment, aripiprazole is a relevant treatment option: |  |
|  | a) in association with the current SGA | **Consensus**: disagreement |
|  | b) as a switch option for the current SGA | No agreement |
| 5. | In the management of an adult patient with schizophrenia presenting with blunted affect associated with **social withdrawal** unimproved by current SGA treatment, aripiprazole is a relevant treatment option: |  |
|  | a) in association with the current SGA | No agreement |
|  | b) as a switch option for the current SGA | **Consensus**: agreement |
|  | **Second round** |  |
| 1. | In the management of an adult patient with schizophrenia presenting with blunted affect associated with **other negative symptoms** partially responsive to the current SGA, addition of aripiprazole in combination with the antipsychotic is a more relevant treatment option than aripiprazole in monotherapy. | **Consensus**: disagreement |
| 2. | In the management of an adult patient with schizophrenia presenting with blunted affect associated with **depressed mood** partially responsive to the current SGA, aripiprazole is a relevant treatment after the failure of combining the current SGA with an antidepressant. | **Consensus**: agreement |
|  | **DOMAIN 2: PREGNANCY** |  |
| N° | **First round** |  |
| 1 | Maintenance of aripiprazole is an appropriate option in a well-controlled adult patient planning a pregnancy. | Trend to agreement |
| 2. | Switching to oral aripiprazole is an appropriate option in a well-controlled adult patient planning a pregnancy. | No agreement |
| 3a. | Maintenance of oral aripiprazole is an appropriate option in a well-controlled adult patient who discovers her pregnancy during the first trimester. | **Consensus**: agreement |
| 3b. | Maintenance of depot aripiprazole is an appropriate option in a well-controlled adult patient who discovers her pregnancy during the first trimester. | No agreement |
| 4. | Switching from the current antipsychotic to aripiprazole is an appropriate option in a well-controlled pregnant patient who develops gestational diabetes. | No agreement |
| 5. | Maintenance of oral aripiprazole is an appropriate option in an adult patient throughout the duration of her pregnancy right up to delivery. | **Consensus**: agreement |
|  | **Second round** |  |
| 1a. | In an adult patient well-controlled on oral aripiprazole who finds herself pregnant, aripiprazole treatment should be maintained. | **Consensus**: agreement |
| 1b. | In an adult patient well-controlled on oral aripiprazole who is planning a pregnancy, aripiprazole treatment should be maintained. | **Consensus**: agreement |
| 2a. | In an adult patient well-controlled on depot aripiprazole who finds herself pregnant, treatment should be switched to the oral formulation of aripiprazole. | Trend to disagreement |
| 2b. | In an adult patient well-controlled on depot aripiprazole who is planning a pregnancy, aripiprazole treatment should be switched to the oral formulation of aripiprazole. | **Consensus**: Disagreement |
|  | **DOMAIN 3: COGNITIVE DYSFUNCTION** |  |
| N° | **First round** |  |
| 1. | In the management of an adult patient with schizophrenia, aripiprazole is a relevant treatment option to preserve cognitive function. | **Consensus**: agreement |
| 2. | In the management of an adult patient with schizophrenia, aripiprazole is a relevant treatment option to improve cognitive problems attributable to the underlying mental disorder. | Trend to agreement |
| 3. | In the management of an adult patient with schizophrenia aripiprazole is more effective than other SGAs for treating cognitive problems attributable to the underlying mental disorder. | No agreement |
| 4. | In the management of an adult patient with schizophrenia, aripiprazole is more effective than other SGAs with respect to the patient’s capacity for social interactions. | Trend to agreement |
| 5. | In the management of an adult patient with schizophrenia presenting with cognitive problems appearing during treatment with the current antipsychotic, addition of aripiprazole is a relevant treatment option if the patient is: |  |
|  | 1. treated with an SGA | No agreement |
|  | 1. treated with an FGA | No agreement |
| 6. | In the management of an adult patient with schizophrenia presenting with cognitive problems appearing during treatment with the current antipsychotic, a switch to aripiprazole is a relevant treatment option if the patient is: |  |
|  | 1. treated with an SGA | Trend to agreement |
|  | 1. treated with an FGA | **Consensus**: agreement |
| 7. | The effectiveness of aripiprazole with respect to cognitive function is dose-dependent (within the approved dose-range) | No agreement |
|  | **Second round** |  |
| 1. | In the management of an adult patient with schizophrenia, addition of aripiprazole in combination with the current antipsychotic is a relevant treatment option in order to improve cognitive problems attributable to the underlying mental disorder. | Trend to disagreement |
| 2. | In the management of an adult patient with schizophrenia presenting with cognitive problems appearing during treatment with the current antipsychotic, a switch to aripiprazole is a relevant treatment option. | **Consensus**: agreement |
|  | **DOMAIN 4: ADDICTIVE COMORBIDITY** |  |
| N° | **First round** |  |
| 1 | Aripiprazole is an appropriate option in an adult with schizophrenia and a comorbid addictive disorder. | **Consensus**: agreement |
| 2a. | Aripiprazole (oral formulation) is an appropriate option as part of a detoxification strategy. | Trend to agreement |
| 2b. | Aripiprazole (depot formulation) is an appropriate option as part of a detoxification strategy. | Trend to agreement |
| 3. | Aripiprazole reduces craving in adult patients with schizophrenia and comorbid addictive disorders. | No agreement |
| 4. | Aripiprazole is the antipsychotic treatment of choice in the context of a methadone substitution therapy. | No agreement |
| 5. | Aripiprazole is an appropriate option for treatment of a first psychotic episode in a patient with a comorbid cannabis use disorder. | **Consensus**: agreement |
| 6. | Aripiprazole is an appropriate option in an adult with schizophrenia and a comorbid alcohol use disorder. | Trend to agreement |
| 7. | Aripiprazole is an appropriate option in an adult with schizophrenia and a comorbid nicotine use disorder. | No agreement |
|  | **Second round** |  |
| 1. | In an adult patient well-controlled on another antipsychotic who has an unresolved addictive disorder, add-on aripiprazole treatment is an appropriate option. | **Consensus**: disagreement |
| 2a. | In an adult patient not controlled on another antipsychotic who has an unresolved addictive disorder, switching to aripiprazole treatment is a relevant therapeutic alternative. | **Consensus**: agreement |
|  | **DOMAIN 5: AUGMENTATION THERAPY FOR CLOZAPINE RESISTANCE** |  |
| N° | **First round** |  |
| 1. | In the management of an adult patient with schizophrenia with clozapine resistance, aripiprazole is a relevant treatment option: |  |
|  | a) in first-line | Trend to agreement |
|  | b) following failure of electroconvulsive therapy (ECT) | No agreement |
| 2. | In the management of an adult patient with schizophrenia with clozapine resistance, aripiprazole is a relevant treatment option: |  |
|  | a) if positive symptoms persist | No agreement |
|  | b) if negative symptoms persist | **Consensus**: agreement |
|  | c) if depressive symptoms persist | No agreement |
| 3. | In the management of an adult patient with schizophrenia with clozapine resistance, augmentation with aripiprazole allows: |  |
|  | a) a reduction of metabolic side-effects | No agreement |
|  | b) a reduction of sedation | No agreement |
| 4. | In the management of an adult patient with schizophrenia with clozapine resistance, a long-acting depot formulation of aripiprazole is a relevant treatment option: | Trend to agreement |
|  | **Second round** |  |
| 1. | In the management of an adult patient with schizophrenia presenting a partial response to clozapine, aripiprazole is a relevant treatment option: | **Consensus**: agreement |

Additional Table 4 Examples of verbatim from the Delphi survey

| **Persistent negative symptoms**   - I would tend to add another class of psychotropic drug specifically to treat anxiety; for the blunted affect, aripiprazole seems indicated to me. - If the negative symptoms are secondary, there may be an interest in combination therapy, but if they are primary, I would switch. - I would rather prescribe an antidepressant as first line rather than changing the antipsychotic treatment in case of persistent mood symptoms. - In patients with persistent negative symptoms, antipsychotic monotherapy is to be preferred. |
| --- |
| **Pregnancy**   - I would keep the current treatment (aripiprazole or other) throughout pregnancy if the patient is well-controlled and the treatment is compatible with a pregnancy. - In pregnant women, I try and use aripiprazole at the minimal effective dose and with monitoring of possible adverse effects in the newborn. - Don’t use depot formulations during pregnancy, unless necessary – in case you need to stop. - I would consider aripiprazole in a woman who is planning a pregnancy, but we have more experience with other SGAs such as risperidone or quetiapine. |
| **Cognitive dysfunction**   - Aripiprazole has a very good cognitive effect, it’s useful for creative patients (such as artists). - Improvement in cognitive function is mostly a result of cognitive remediation - you need to propose cognitive remediation as well as the antipsychotic. - It is mainly the antipsychotic efficacy of aripiprazole, with as little sedation as possible, that allows behavioral management and cognitive remediation to be implemented. - In case of persistent cognitive symptoms on the current SGA, I would not add aripiprazole, I would switch, to limit the side effects. |
| **Addictive comorbidity**   - Unfortunately, aripiprazole does not always reduce craving, in my experience. - If the patient is well controlled with another SGA, I would think twice before switching, but if addictive behavior features as a symptom in an insufficiently controlled patient, I would consider switching to aripiprazole. - Aripiprazole would be my first choice in first episode schizophrenia with a cannabis use problem. - In a patient with an alcohol use disorder, you need to simultaneously manage the addiction. |
| **Augmentation therapy for clozapine resistance**   - Aripiprazole has a pharmacodynamic profile complementary to that of clozapine. - Aripiprazole would not be expected to improve the deleterious metabolic effects of clozapine, since it is co-prescribed. - Importance of a blood test and optimising the dose of clozapine before moving to an augmentation therapy. - In case of persistent negative symptoms, I try to stay in the low therapeutic zone of clozapine. In case of residual depression, I treat the depression. |

Additional Figure 1 PRISMA flow diagram for the literature search


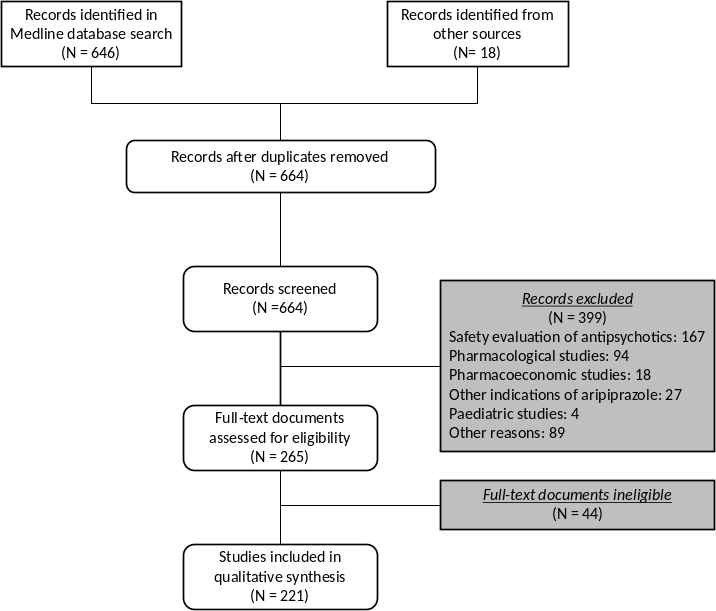

Supplement: Supplementary file 1 — Additional file 1: Place of the partial dopamine receptor agonist aripiprazole in themanagement of schizophrenia in adults: a Delphi consensus study. Additional Table 1. Evidence level classification of the Frenchhealth authorities. Additional Table 2. Studies identified in the literature review. Additional Table 3. Questions asked in the Delphisurvey. Additional Table 4. Examples of verbatim from theDelphi survey. Additional Figure 1. PRISMAflow diagram for the literature search. [file 12888_2022_4008_MOESM1_ESM.docx]
